# Supplementary material for: A parametrically programmable delay line for microwave photons
Source: Nat Commun. 2024 May 31;15:4640. doi: 10.1038/s41467-024-48975-x (PMC11143279; doi:10.1038/s41467-024-48975-x)
Supplement: Supplementary file 1 — Supplementary Information [file 41467_2024_48975_MOESM1_ESM.pdf]

Supplementary Information for: A parametrically programmable delay line for microwave photons

Takuma Makihara,[1,*] Nathan Lee,[1] Yudan Guo,[1] Wenyan Guan,[1] and Amir Safavi-Naeini[1,†]

[1]*Department of Applied Physics, Stanford University, Stanford, California 94305, USA*

(Dated: May 12, 2024)

SUPPLEMENTARY NOTE 1: DEVICE PARAMETERS

Our device parameters are summarized in Supplementary Table 1. To determine φ_k for the CPW resonators and φ_b for the buffer mode, we simulate the capacitive coupling between the CPWs and the buffer and fit the inductance of the ATS junction array that reproduces the measured buffer mode frequency. We leave the ATS junction array and SQUID junctions open in our capacitance simulation. We find the ATS junction array inductance to be $L_b = 7.50$ nH, corresponding to $E_{L_b}/h = 21.8$ GHz. This values agrees within 8% of values computed from room-temperature resistance measurements (using the Ambegaokar-Baratoff formula) of nominally identical junctions that are fabricated near the device. We use an array of 21 junctions to form the ATS junction array. The SQUID junction energy is also inferred from room-temperature measurements. An ATS that was fabricated in parallel with the measured device is pictured in Supplementary Fig. 1.

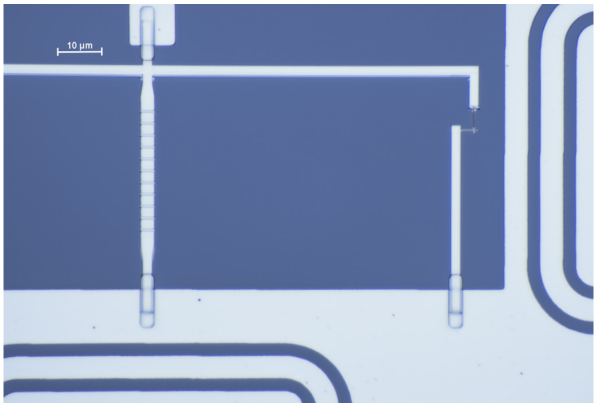

SUPPLEMENTARY FIG. 1. Optical micrograph of the ATS. This is an image of an identical device that was fabricated in parallel with the measured device, with the exception that the Dolan-style junctions in the ATS superinductor are nominally 0.975 μm narrower.

Given the capacitance matrix, the ATS inductance, and by treating the quarter-wavelength CPW resonators as effective lumped element LC resonators (based on their designed characteristic impedances and lengths), we can diagonalize the circuit to find the eigenfrequencies and ZPFs at the ATS node. We find that our calculated eigenfrequencies agree well with our measured frequencies (within 1%). The resulting eigenvectors are used to compute φ_b and φ_k.

The buffer mode and the CPW modes are characterized using spectroscopy with a vector network analyzer when the buffer is flux biased at the saddle point. We are able to directly measure the CPW modes due to parasitic capacitances between the CPW resonators and the readout transmission line. The measured values are reported in Supplementary Table 1. The buffer frequency, extrinsic loss, and intrinsic loss are respectively labeled by ω_b, $\kappa_{b,e}$, $\kappa_{b,i}$. The reported uncertainties are standard deviations from repeatedly measuring the buffer over 95 samples. The CPW frequencies, extrinsic losses (due to parasitic capacitance to the readout line), intrinsic losses, and intrinsic quality factors are respectively labeled by ω_k, $\kappa_{k,e}$, $\kappa_{k,i}$, and $Q_{i,k}$. The reported uncertainties are standard deviations from repeatedly measuring the CPWs over 81 samples, all at single-photon powers.

SUPPLEMENTARY NOTE 2: EXPERIMENTAL DETAILS AND SETUP

The experimental setup is shown in Supplementary Fig. 2. Here, we describe the main components of our experiment: (1) parametric driving of the buffer mode, (2) playing and digitizing pulses, (3) DC flux biasing, and (4) flux pumping.

Parametric driving of the buffer mode: We use a 5 GS/s AWG to simultaneously play the seven drive tones that parametrically couple the CPW resonators to the buffer mode. Each individual drive tone had a V_{pp} ranging from 3.3 mV to 27 mV depending on the detuning. These tones are amplified after up-conversion to provide strong enough driving on the buffer mode. The output is combined with the output of the QM OPX and Octave. The on-chip power of each individual drive tone approximately ranged from −99.6 dBm to −120.0 dBm depending on the detuning.

Playing and digitizing pulses: We use a QM OPX and Octave to generate Gaussian pulses that we store in the PADL. The pulses are synthesized and played from the Analog Outputs on the OPX, which are then upconverted using mixers and local oscillators (LOs) in the Octave. The Octave conveniently calibrates its internal mixers to remove spurious tones from the LO. The up-

* makihara@stanford.edu
† safavi@stanford.edu

$\omega_b/2\pi$	5.0073 GHz $\pm$ 600 kHz
L_b	7.50 nH
φ_b	0.336
$\kappa_{b,e}/2\pi$	3.95 MHz $\pm$ 60 kHz
$\kappa_{b,i}/2\pi$	130 kHz $\pm$ 40 kHz
E_J/h	5.28 GHz

k	$\omega_k/2\pi$ [GHz $\pm$ kHz]	φ_k	$\kappa_{k,e}/2\pi$ [kHz $\pm$ kHz]	$\kappa_{k,i}/2\pi$ [kHz $\pm$ kHz]	$Q_{i,k} \times 10^3$
1	6.904939 $\pm$ 2	0.0186	37 $\pm$ 1	32 $\pm$ 3	220 $\pm$ 20
2	6.975562 $\pm$ 3	0.0228	40 $\pm$ 2	29 $\pm$ 2	240 $\pm$ 20
3	7.156324 $\pm$ 9	0.0210	70 $\pm$ 5	41 $\pm$ 7	170 $\pm$ 30
4	7.247145 $\pm$ 3	0.0175	57 $\pm$ 1	34 $\pm$ 4	210 $\pm$ 30
5	7.318975 $\pm$ 4	0.0179	74 $\pm$ 1	39 $\pm$ 4	190 $\pm$ 20
6	7.389379 $\pm$ 2	0.0186	102 $\pm$ 2	25 $\pm$ 3	300 $\pm$ 40
7	7.460333 $\pm$ 4	0.0211	132 $\pm$ 4	67 $\pm$ 6	110 $\pm$ 10

SUPPLEMENTARY TABLE 1. Table of device parameters.

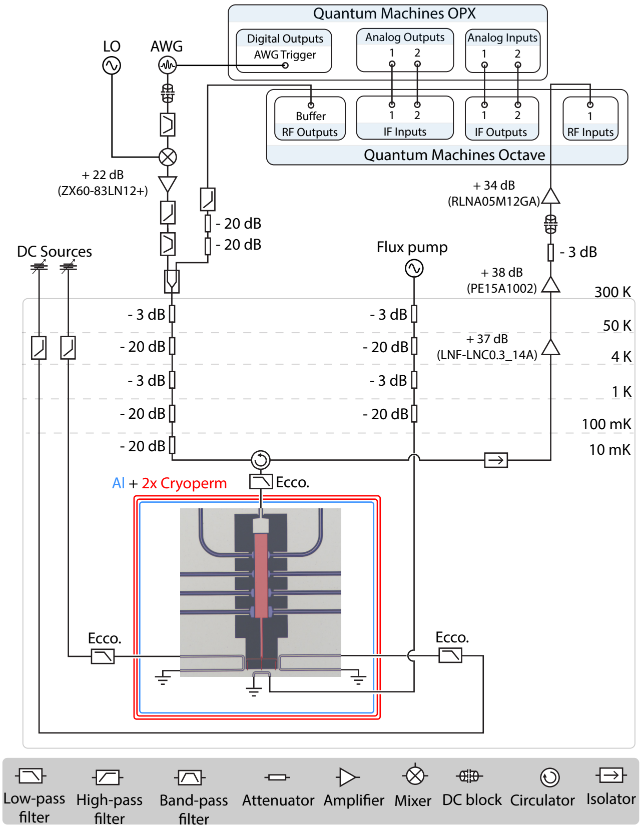

SUPPLEMENTARY FIG. 2. Experimental setup.

converted pulse is played from the RF Outputs on the Octave. Similarly, the Octave down-converts pulses after they interact with our device. Specifically, pulses incident on the RF Inputs are down-converted and played from the IF Outputs, which are then sent to the Analog Inputs on the OPX. For Gaussian pulses with $\langle n \rangle \simeq 1$ and temporal FWHM of 471 ns, the peak power on-chip is approximately -140.3 dBm.

DC flux biasing: As shown in Fig. 1g, we have two flux lines that are symmetrically placed on either side of the ATS. We use two voltage sources (SRS SIM928) to bias these two flux lines. The flux lines are low-pass filtered at the 4 K stage of our dilution refrigerator (Aivon Therma-24G).

Flux pumping: As shown in Fig. 1g, we have one additional flux line that is placed directly underneath the ATS (where the ATS is grounded). This flux line provides a magnetic flux that symmetrically threads both ATS loops, thereby providing the parametric flux pump $\epsilon_p(t)$. This flux pump is sourced from a power signal generator (Keysight E8257D PSG). The flux pump power on-chip is approximately -57.6 dBm.

In Supplementary Fig. 3 we illustrate all the rele-

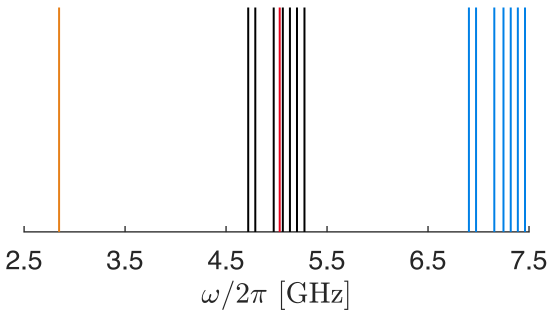

SUPPLEMENTARY FIG. 3. Illustration of relevant frequencies for our experiment. We show the CPW frequencies in blue, the buffer frequency in red, the parametric drive frequencies in black, and the parametric pump frequency in orange.

vant frequencies for our experiment. We show the CPW frequencies in blue (from Supplementary Table 1) and the buffer frequency in red (from Supplementary Table 2). We also show the parametric drive frequencies in black, which are (in GHz): 4.719403, 4.789553, 4.971818, 5.062160, 5.132469, 5.202372, 5.276428. Finally, we show the pump frequency in orange, which is 2.84654 GHz.

SUPPLEMENTARY NOTE 3: DC FLUX BIASING

We use the two DC flux lines to find the so-called saddle point where $\varphi_\Sigma = \varphi_\Delta = \pi/2$. In Supplementary Fig. 4, we show the frequency of the buffer mode in the vicinity of the saddle point as we sweep our two voltage supplies. We work in the basis of $V_\Sigma = (V_1 + V_2)/2 \propto \varphi_\Sigma$ and $V_\Delta = (V_1 - V_2)/2 \propto \varphi_\Delta$, where V_1 and V_2 are the voltages of the individual voltage supplies.

In reality, slight junction asymmetry between the two SQUID junctions can lead to slight differences in the buffer frequency at different saddle points given by $\varphi_{\Sigma,\Delta} = \pm\pi/2$. In Supplementary Fig. 4a and 4b, we show two different saddle points taken at two different values of φ_Σ. Fortunately, we find these two saddle point frequencies agree within a linewidth of the buffer mode. We also confirmed this at saddle points taken at two different values of φ_Δ. Therefore, we neglect contributions from junction asymmetry in this work.

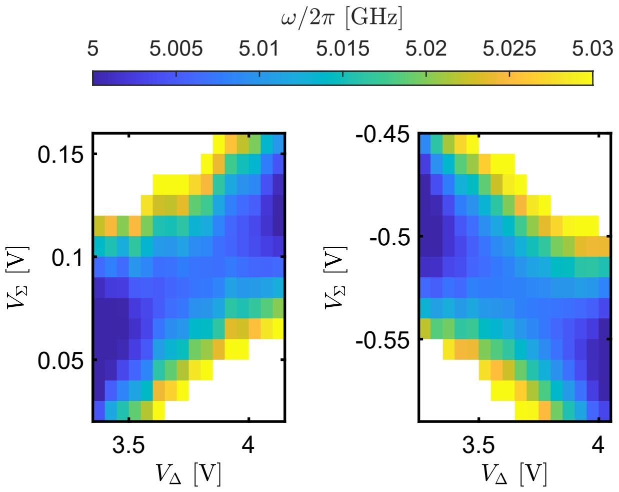

SUPPLEMENTARY FIG. 4. Finding the saddle point. We tune the buffer mode frequency by sweeping our two voltage sources. The point where $\varphi_\Sigma = \varphi_\Delta = \pi/2$ is easily identified as a saddle point in a color map of the mode. Here, we show saddle points at two different values of V_Σ.

SUPPLEMENTARY NOTE 4: PARAMETRIC DELAY LINE PARAMETERS

The scattering parameters in Fig. 2a of the main text are fit to the following model S_{11} model:

$$S_{11}(\omega) = 1 - \frac{\kappa_{b,e}}{i(\omega_b - \omega) + \frac{\kappa_b}{2} + i\sum_k \frac{|g_k|^2}{\omega - \omega_k' + i\kappa_k/2}} \quad (1)$$

where ω_b is the buffer frequency, $\kappa_{b,e}$ is the buffer extrinsic loss rate, $\kappa_b = \kappa_{b,e} + \kappa_{b,i}$ is the total loss rate of the buffer (including extrinsic and intrinsic loss), g_k is the parametric coupling between the buffer and the kth CPW, ω_k' is the frequency of the photons from the kth CPW that are being parametrically coupled to the buffer, and κ_k is the total loss rate of the kth CPW.

The fit parameters are listed in Supplementary Table 2. In the first column, we list the CPW frequencies from Supplementary Table 1 to index each row. The reported uncertainties are standard deviations from repeatedly measuring $S_{11}(\omega)$ 500 times overnight and fitting the parameters.

SUPPLEMENTARY NOTE 5: DOMINANT SOURCES OF INFIDELITY AND ADDITIONAL SIMULATIONS

Given two temporal modes that are defined by annihilation operators $\hat{A}_1 = \int \mathrm{d}t f(t)\hat{a}_{\text{wg}}(t)$ and $\hat{A}_2 = \int \mathrm{d}t g(t)\hat{a}_{\text{wg}}(t)$, we define the fidelity as $F = |\int dt f^*(t)g(t)|^2$ [2]. This is the fidelity associated with two single-photon wavepackets $\hat{A}_1^\dagger|\text{vac}\rangle$ and $\hat{A}_2^\dagger|\text{vac}\rangle$. Even though we are not encoding quantum information in single-photon wavepackets, we can still estimate this fidelity from the detected field, i.e., the complex digitized data collected by the analog-to-digital converter (ADC). We first turn off the pumps and tune the buffer mode off resonance. The device acts as a mirror which reflects the input pulse. Our ADC records the reflected signal and averages the measured field to find $V_{\text{in}}[t] = I_{\text{in}}[t] + iQ_{\text{in}}[t]$, corresponding to the mean field in the reflected pulse. We then operate the PADL in the delay line mode, and record $V_{\text{out}}[t] = I_{\text{out}}[t] + iQ_{\text{out}}[t]$, corresponding to the mean field of delayed pulse. We normalize both of these averaged traces by the same constant such that $\sum_t V_{\text{in}}[t]V_{\text{in}}[t]^* = 1$. Fidelity for a given delay τ is then calculated by $F[\tau] = \left(\sum_t V_{\text{in}}[t-\tau]V_{\text{out}}[t]^*\right)^2$. We find that fidelity is maximized for $\tau \simeq T_{\text{rt}}$, so that $F = \max_\tau(F[\tau])$ which is very nearly $F[T_{\text{rt}}]$. Note that in the above formulation, since we are using the same normalization constant for both the input and output fields, the fidelity captures the effects of both loss and distortion.

We can simulate this whole process by numerically integrating the equations of motion for $\hat{a}(t)$ and $\hat{b}(t)$ derived from the Hamiltonian in Eq. 4 of the main text. This

$\omega_b/2\pi$ [GHz ± kHz]	5.03123 ± 70
$\kappa_{b,e}/2\pi$ [MHz ± kHz]	3.37 ± 70
$\kappa_{b,i}/2\pi$ [kHz ± kHz]	440 ± 60

$\omega_k/2\pi$ [GHz]	$\omega'_k/2\pi$ [GHz ± kHz]	$\kappa_k/2\pi$ [kHz ± kHz]	$g_k/2\pi$ [kHz ± kHz]
6.904939	5.032140 ± 1	67 ± 6	530 ± 20
6.975562	5.032625 ± 4	78 ± 8	530 ± 20
7.156324	5.031112 ± 9	109 ± 7	540 ± 10
7.247145	5.031607 ± 4	90 ± 8	560 ± 10
7.318975	5.033132 ± 5	130 ± 10	520 ± 20
7.389379	5.033617 ± 4	140 ± 20	520 ± 10
7.460333	5.03058 ± 10	210 ± 30	520 ± 20

SUPPLEMENTARY TABLE 2. Table of fit parameters in $S_{11}(\omega)$.

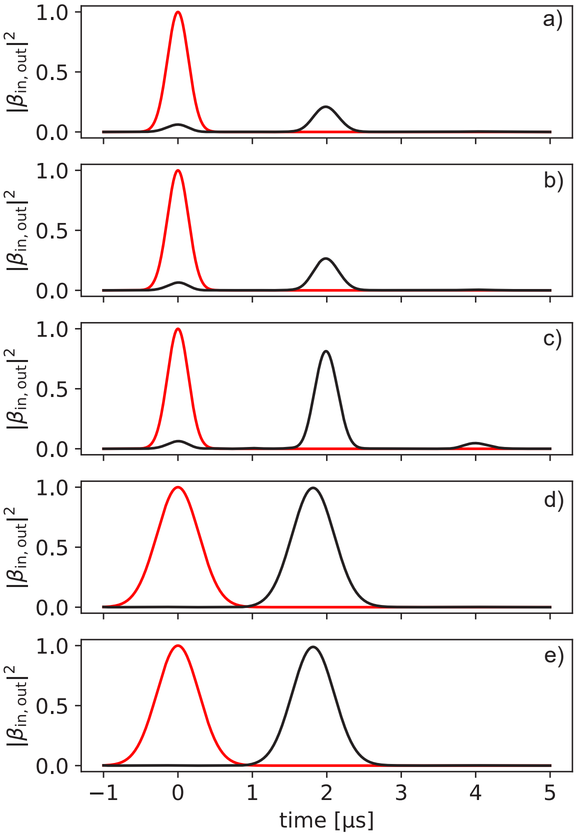

SUPPLEMENTARY FIG. 5. Fidelity loss channels. (a) Simulated input (red) and output (black) pulses for the Hamiltonian parameters from our experiment as reported in Supplementary Table 2 and with an input Gaussian pulse (temporal FWHM of 471 ns). In (b) and (c) we run identical simulations but with $\kappa_{b,i} = 0$ and $\kappa_{b,i} = \kappa_k = 0$, respectively. In (d) we not only consider no intrinsic loss, but we also fix Δ_k to be evenly spaced from $-\kappa_{b,e}/2$ to $+\kappa_{b,e}/2$ over 7 modes and fix $g/2\pi = 562.4$ kHz. In (e) we consider the same delay line as in (d) but consider nonzero κ_k given by state-of-the-art CPW resonators quality factors [1].

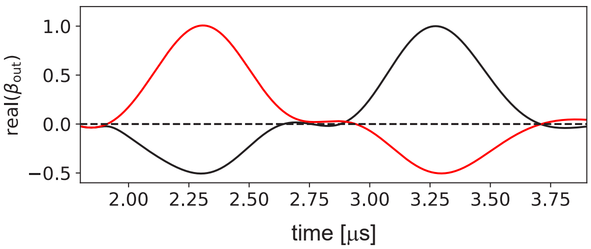

SUPPLEMENTARY FIG. 6. Additional pulse-swapping simulation. Simulation of two delayed pulses for a lossless delay line where the parametrically converted CPW photon detunings Δ_k are not swapped (i.e. $\Delta_k \to \Delta_k$, shown in red) and when the detunings are swapped (i.e. $\Delta_k \to -\Delta_k$, shown in black). The two pulses have a relative π phase shift and the earlier pulse has an amplitude that is twice as large as the later pulse.

allows us to estimate the dominant sources of fidelity loss in our delay line. The equations of motion are given by:

$$\begin{aligned}\frac{d}{dt}\hat{a}_k &= -i\Delta_k\hat{a}_k - ig_k^*\hat{b} - \frac{\kappa_k}{2}\hat{a}_k - \sqrt{\kappa_{k,i}}\hat{a}_{\mathrm{in,i}} \\ \frac{d}{dt}\hat{b} &= -\frac{\kappa_b}{2}\hat{b} - i\sum_k g_k\hat{a}_k - \sqrt{\kappa_{b,e}}\hat{b}_{\mathrm{in}}\end{aligned} \quad (2)$$

Since these differential equations are linear, we can take the expected values of the fields, and obtain exact mean field equation for $\alpha_k = \langle\hat{a}_k\rangle$ and $\beta = \langle\hat{b}\rangle$. The input and output photon fluxes incident on the buffer mode can be obtained from the input-output boundary condition $\beta_{\mathrm{out}} = \beta_{\mathrm{in}} + \sqrt{\kappa_{b,e}}\beta$.

In Supplementary Fig. 5a, we plot the input photon flux in red and output photon flux in black for different parametric delay line parameters. In the top figure, we simulate the Hamiltonian parameters from our experiment, which are reported in Supplementary Table 2. We choose $\beta_{\mathrm{in}}(t)$ to be a Gaussian pulse with temporal

FWHM of 471 ns to match the experiment. The black trace agrees well with the results measured by our ADC in Fig. 2 of the main text, and we calculate a state fidelity of $F = 0.24$. For our fidelity calculation, we normalize the input mode profile $(f(t))$ such that $\int \mathrm{d}t|f(t)|^2 = 1$, and we normalize the delayed mode profile $(g(t))$ by the same factor. In Supplementary Fig. 5b, we set $\kappa_{b,i} = 0$ and find that the fidelity improves to $F = 0.31$. In Supplementary Fig. 5c, we set all intrinsic loss channels to zero i.e. $\kappa_{b,i} = \kappa_k = 0$ and find that the fidelity improves to $F = 0.86$. In Supplementary Fig. 5d, we consider a lossless delay line where the Hamiltonian parameters are chosen such that Δ_k is evenly spaced from $-\kappa_{b,e}/2$ to $+\kappa_{b,e}/2$ over 7 modes and fix $g/2\pi = 562.4$ kHz (roughly corresponding to a frequency comb with finesse = 1.5). We also increase the pulse temporal FWHM to 942 ns, which improves the fidelity to $F = 0.996$. Finally, in Supplementary Fig. 5e, we consider the same delay line parameters as in Supplementary Fig. 5d but we add CPW loss again. We consider state-of-the-art CPW quality factors [1] with Q_i of $15e6$ and find that $F = 0.991$.

In Supplementary Fig. 5a, we also observe a small reflected pulse at $t = 0$ in the parametrically delayed (black) trace. This small reflected pulse was present throughout our experiments. This is due to the slight impedance mismatch that arises from our non-ideal phase response. We observe significant reduction in this reflected pulse when we optimize our Hamiltonian parameters and increase our pulse bandwidth, as can be seen in Supplementary Fig. 5d. One challenge we faced in preparing a perfect parametric delay line (i.e. a perfectly linear phase response) is the challenge of rapidly and reliably fitting $S_{11}(\omega)$ while tuning the parametric drive amplitudes and detunings, especially given the large number of parameters to fit. Another source of reflections at $t = 0$ could be inevitable impedance mismatches in our device packaging, most notably from the wirebond connecting our readout line to the PCB.

In Supplementary Fig. 6, we simulate the experiment where we control the detunings of the parametrically converted CPW photons Δ_k to swap two pulses in time. We consider a lossless delay line (with parameters identical to those in Supplementary Fig. 5d) and we consider two pulses with temporal FWHM = 377 ns and separation 1000 ns. In addition to a relative π phase shift between the two pulses, we have the first pulse have an amplitude that is twice as large as the second pulse. The red trace in Supplementary Fig. 6 plots the pulse when the detunings have not been swapped (i.e. $\Delta_k \to \Delta_k$), and the black trace plots the pulse when the detunings have been swapped (i.e. $\Delta_k \to -\Delta_k$). We clearly see that swapping the detunings swaps the pulse in time.

We can use these simulations to approximate the minimum number of resonators needed to perform the simplest possible experiment: delaying a pulse by more than its temporal FWHM. The figure of merit with delay lines is the delay-bandwidth product (DBP). Assuming Δ_k is evenly spaced from $-\kappa_b/2$ to $+\kappa_b/2$, we have that the DBP $= N - 1$ for the PADL, where N is the number of resonators that are being parametrically coupled to the buffer. The narrowest temporal pulse that one could use will have a temporal FWHM $\tau_{\mathrm{FWHM}} \simeq 2\pi/\kappa_b$. Therefore, we have that $\tau_{\mathrm{delay}}/\tau_{\mathrm{FWHM}} \simeq N - 1$. To visually differentiate the stored pulse from the delayed pulse, we require $2\tau_{\mathrm{FWHM}} \lessapprox \tau_{\mathrm{delay}}$ and thus one would require a bare minimum of 3 resonators.

SUPPLEMENTARY NOTE 6: GAIN, ATTENUATION, AND ADDED NOISE CALIBRATION

To bound the number of added noise photons caused by strongly driving our device, we first need to carefully calibrate the gain of our measurement apparatus. To do this, we need an in-situ noise source, i.e. some way to relate the number of on-chip quanta to a measurable value at room-temperature.

In our system, we leverage the three-wave mixing interaction of the ATS to operate our CPW resonators as MPOs. We use the number of resonator photons n near threshold as our in-situ noise source. The smooth increase in n vs. driving field observed in our quantum MPO is similar to the behaviour of a laser near threshold [3–5].

To operate the kth CPW as an MPO, we pump our device at a frequency $\omega_p = 2\omega_k - \omega_b$ and drive our buffer at a frequency $\omega_d = \omega_b$. To find where the pump and drive frequencies precisely satisfy these energy conservation relations, we sweep them and measure the spectrum of the CPW resonator. Far below threshold, n is only nonzero if the pump and drive frequencies are resonant [6].

Once we have determined our resonant pump and drive frequencies, we measure the spectrum of the CPW resonator at different drive strengths. We fit the integrated

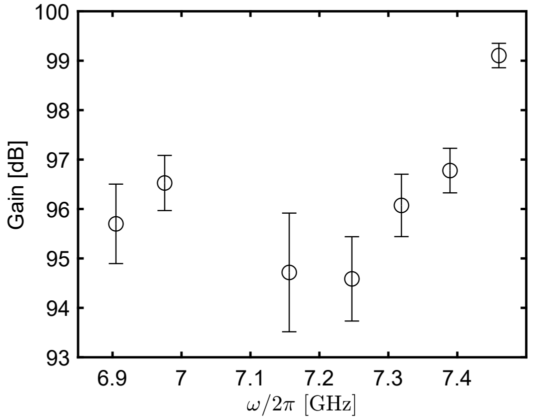

SUPPLEMENTARY FIG. 7. Fitted gain at the CPW frequencies from operating them as quantum MPOs near threshold.

$\omega_k/2\pi$ [GHz]	Gain [dB]	κ_2/κ_1	$\langle n\rangle$
6.904939	95.70	8.406e-4	0.143
6.975562	96.53	22.09e-4	0.092
7.156324	94.72	6.323e-4	0.109
7.247145	94.59	5.523e-4	0.143
7.318975	96.07	7.165e-4	0.083
7.389379	96.78	12.73e-4	0.065
7.460333	99.11	70.65e-4	0.021

SUPPLEMENTARY TABLE 3. Table of the fitted gain, κ_2/κ_1, and added noise.

PSD vs. the drive strength to a master equation model with Hamiltonian $\hat{H}$ and jump operators $\hat{L}_1$ and $\hat{L}_2$ given by

$$\begin{aligned} \hat{H} &= i\epsilon_2\hat{a}^2 + \text{h.c.}, \\ \hat{L}_2 &= \sqrt{\kappa_2}\hat{a}^2, \\ \hat{L}_1 &= \sqrt{\kappa_1}\hat{a}, \end{aligned} \tag{3}$$

where ϵ_2 is the two-photon drive strength, κ_2 is the two-photon loss rate, and κ_1 is the single-photon loss rate. An example for the mode at 6.975562 GHz is shown in Fig. 4a of the main text.

Our only fit parameters are: (1) the proportionality constant between ϵ_2/κ_1 and the driving field, (2) the proportionality constant between n and the integrated PSD, which is related to the gain, and (3) κ_2/κ_1. By normalizing everything with respect to κ_1, our fits are robust to pump-induced effects that may worsen κ_1. In Supplementary Fig. 7, we plot our measured gain (in dB) at the different CPW frequencies (error bars are the standard deviation from repeated measurements and fits). The gain is the proportionality constant between the integrated PSD at room temperature and the power leaking out of the MPO ($P_{\text{MPO},k} = \hbar\omega_k\bar{n}\kappa_{k,e}$). We also report our fitted gains and κ_2/κ_1 in Supplementary Table 3.

With the gain calibrated near the CPW frequencies, we can bound the noise added from strongly driving our device. Specifically, we turn on the parametric pump and drives that we use for our delay line experiments while measuring the CPW spectra. An example of such a spectrum is shown in Fig. 4b. In Supplementary Table 3 we report the added noise in each CPW mode and observe it to be much less than 1 photon per mode. In the first column, we list the CPW frequencies from Supplementary Table 1 to index each row.

[1] K. D. Crowley, R. A. McLellan, A. Dutta, N. Shumiya, A. P. Place, X. H. Le, Y. Gang, T. Madhavan, M. P. Bland, R. Chang, *et al.*, Disentangling losses in tantalum superconducting circuits, Physical Review X **13**, 041005 (2023).

[2] K. M. Gheri, K. Ellinger, T. Pellizzari, and P. Zoller, Photon-wavepackets as flying quantum bits, Fortschritte der Physik: Progress of Physics **46**, 401 (1998).

[3] G. Bjork and Y. Yamamoto, Analysis of semiconductor microcavity lasers using rate equations, IEEE Journal of Quantum Electronics **27**, 2386 (1991).

[4] G. Björk, A. Karlsson, and Y. Yamamoto, Definition of a laser threshold, Physical Review A **50**, 1675 (1994).

[5] P. R. Rice and H. Carmichael, Photon statistics of a cavity-qed laser: A comment on the laser–phase-transition analogy, Physical Review A **50**, 4318 (1994).

[6] C. Berdou, A. Murani, U. Reglade, W. C. Smith, M. Villiers, J. Palomo, M. Rosticher, A. Denis, P. Morfin, M. Delbecq, *et al.*, One hundred second bit-flip time in a two-photon dissipative oscillator, PRX Quantum **4**, 020350 (2023).
